# Synthesis of Vinyl-Containing Polydimethylsiloxane in An Active Medium

**DOI:** 10.3390/polym16020257

**Published:** 2024-01-16

**Authors:** Alina G. Khmelnitskaia, Aleksandra A. Kalinina, Ivan B. Meshkov, Rinat S. Tukhvatshin, Georgii V. Cherkaev, Sergey A. Ponomarenko, Aziz M. Muzafarov

**Affiliations:** Enikolopov Institute of Synthetic Polymeric Materials of Russian Academy of Sciences (ISPM RAS), Profsoyuznaya 70, 117393 Moscow, Russia; alina.khmelnitskaya@ispm.ru (A.G.K.); ivanbm@ispm.ru (I.B.M.); ponomarenko@ispm.ru (S.A.P.); aziz@ispm.ru (A.M.M.)

**Keywords:** active medium, polycondensation, methylvinylsiloxanes, thermal condensation, microstructural analysis

## Abstract

This research deals with the synthesis of copoly(methylvinyl)(dimethyl)siloxanes by the copolycondensation of dimethyldiethoxy- and methylvinyldimethoxysilane in an active medium, followed by thermal condensation in a vacuum. We achieved a range of copolymers exhibiting finely tuned molecular weights spanning between 1500 and 20,000 with regulated functional methylvinylsiloxane units. Analysis of the microstructure showed that the copolymerization predominantly formed products demonstrating a random distribution of units (R~1). However, an increase in the content of vinyl-containing monomers increases the R parameter, indicating an enhanced tendency towards alternating linkages within the copolymer matrix.

## 1. Introduction

Polysiloxanes are extensively applied in many fields, including the cosmetic industry, electronics, biomedicine, artificial muscles, etc. [1,2,3,4,5,6,7]. Such a diversity of applications is owing to the unique properties of polysiloxanes: a wide range of working temperatures, weather resistance, inertness, hydrophobicity, chemical stability, low dielectric permittivity, etc. Among polysiloxanes, polydimethylsiloxanes (PDMSs) are renowned and form the base for silicone sealants, compounds, and rubbers [8,9,10,11]. To enhance PDMS properties, various modifying groups are incorporated into the main chain. For instance, phenyl substituents increase heat resistance and allow adjustment of optical properties via refractive index alterations. Introducing ethyl substituents decreases the glass transition temperature [12,13], while polar substituents enhance dielectric permittivity and intermolecular interaction [14,15,16,17,18,19,20]. Hydride functional groups in polysiloxane main chains may serve various purposes, including utilization in hydrophobicity, in polyadditional compositions as crosslinking agents [21,22], and alongside vinyl-containing polysiloxanes as matrices for polymeranalogical transformations [23,24]. Hydrothiolation, entailing the addition of thiols to substitutes containing unsaturated bonds, has gained substantial traction in polymer modification [14,25,26,27,28,29,30,31]. This process offers advantages, including a low activation energy, diverse radical formation sources, high yields for the target product (primarily β-addition products), and minimal by-products. Therefore, the ultimate structure, composition, and properties of the polysiloxanes modified with hydrothiolation depend significantly on the initial structure of the matrix, as well as the number and placement of unsaturated vinyl or allyl groups in the polymer chain.

The main industrial method for obtaining vinyl-containing PDMS involves the hydrolytic polycondensation of dimethyl- and methylvinyldichlorosilanes followed by the catalytic rearrangement of the hydrolysis product [32]. However, this method presents complexities in controlling product composition, generates hydrochloric acid waste, and requires additional purification stages, including the stage of purification of the target copolymer from the equilibrium amount of cyclosiloxanes (10–15%) formed during the second stage. An alternative method for the preparation of vinyl-containing siloxanes is the ring-opening equilibrium copolymerization of octamethylcyclotetrasiloxane and 1,3,5,7–tetramethyl, 1,3,5,7–tetravinylcyclotetrasiloxane [33]. This method makes it possible to obtain copolymers with specified molecular weight characteristics and the content of vinyl groups, but it is also characterized by the presence of a stage of purification of the polymer from cycles formed as a result of the process equilibrium. Considering the fact that the initial monomers for polymerization—cyclosiloxanes—are also obtained by the polycondensation of functional silanes, it seems a promising task to develop polycondensation approaches to vinyl-containing PDMS synthesis that do not contain cyclic impurities, allowing for the control of the content of vinyl groups and molecular weight characteristics and not requiring equilibration processes.

One of the modern eco-friendly polycondensation methods for the production of polyorganosiloxanes with a given composition and structure is polycondensation in an active medium—an excess of anhydrous acetic acid [32]. In this case, acetic acid is simultaneously a reagent, a catalyst, and a solvent. Unlike the classical catalytic hydrolytic polycondensation of alkoxysilanes, this cascade process occurs in a homogeneous environment, does not require the use of water, since it is generated at one of the stages, and allows the production of polymers of a given composition and structure. Previous studies have shown the possibility of linear dimethyl-, methylphenyl- and methylbenzylsiloxane homopolymers selectively obtained using this method without an additional stage of catalytic rearrangement. This fact makes it promising to expand this approach to linear copolymers, including vinyl-containing PDMS [34,35,36,37].

The purpose of this work was to study the possibilities of the copolycondensation of methylvinyl- and dimethylalkoxysilanes in an active medium to obtain α,ω-dihydroxypoly(methylvinyl)(dimethyl)siloxanes with a controlled molecular weight and content of vinyl groups.

## 2. Materials and Methods

### 2.1. Materials

Unless otherwise stated, all chemicals were reagent-grade and used without purification.

Dimethyldiethoxysilane, methylvinyldimethoxysilane, methyl tertbutyl ether (MTBE) (EKOS-1, Moscow, Russia), acetic acid (EKOS-1, Moscow, Russia), anhydrous sodium sulfate (EKOS-1, Moscow, Russia), 1-decanethiol (ABCR, Karlsruhe, Germany), trimethylchlorosilane 97% (ABCR, Karlsruhe, Germany), pyridine (EKOS-1, Moscow, Russia), and toluene (ECOS-1, Moscow, Russia) were used.

All reagents were purified using standard methods [38]. Before the synthesis, dimethyldiethoxysilane and methylvinyldimethoxysilane were distilled. Acetic acid was dried over P_2_O_5_, distilled, and kept in an inert atmosphere. Pyridine was dried over BaO, distilled, and kept under 3 Å molecular sieves.

### 2.2. Characterization

^1^H nuclear magnetic resonance (NMR) spectra were recorded at room temperature on a Bruker WP 250 SY spectrometer (250.13 MHz) for all samples.

^29^Si NMR spectra were recorded at room temperature on a Bruker Avance II 300 (59.64 MHz) to analyze the microstructure of copolymers. The spectra were processed in MestReNova software (v. 12.0). Chemical shifts (δ) in ppm were calibrated to residual solvent peaks (CDCl_3_: δ = 7.25 ppm).

IR spectra were recorded on an IR Fourier spectrometer—Nicolet iS50 (Thermo Scientific, Waltham, MA, USA)—in the ATR mode with 32 scans for each wave number in the range of 1000–4000 cm^−1^.

Gel permeation chromatograms (GPCs) were recorded using a GPC system, consisting of high-pressure pump Stayer Series II (Akvilon, Podolsk, Russia), refractometric detector RIDK 102 (Laboratory Instruments Prague, Prague, Czech Republic), and column thermostat JETSTREAM 2 PLUS (KNAUER, Berlin, Germany). With temperature control at 40 °C, toluene was used as the eluent, with a flow rate 1 mL/min. Analytical separation was performed using a 7.8 mm × 300 mm Phenomenex column (Spectralab Scientific Inc., Torrance, MA, USA) filled with Phenogel sorbent with a pore size from 500 Å to 10^4^ Å. Molecular masses were determined using polystyrene standards.

Gas–Liquid Chromatography (GLC) analysis was performed on a Chromatek Analytic 5000 chromatograph (CHROMATEC, Yoshkar-Ola, Russia), with a katharometer detector, a helium carrier gas, 2 m × 3 mm columns, and a stationary phase SE-30 (5%), printed on Chromaton-H-AW. Registration and calculation of data were carried out using the program “Chromatek Analyst” (CHROMATEC,Yoshkar-Ola, Russia).

Gas chromatography–mass spectrometry (GC-MS) analysis of the initial compounds was performed on a Shimadzu GCMS-QP2020 Gas Chromatograph Mass Spectrometer with an SH-RTx-5MS column (30 m × 0.25 mm i.d. × 0.25 μm, Shimadzu, Kyoto, Japan), electron impact (EI) ionization method, and single quadrupole detector (positive ions).

### 2.3. Synthesis of PDMS-50-Vin (MeVin/Me_2_ = 1/1) by Simultaneous Mixing

Dimethyldiethoxysilane (3.7 g, 0.025 mol), methylvinyldimethoxysilane (3.3 g, 0.025 mol), and acetic acid (28.6 mL, 0.5 mol) were added simultaneously to a 100 mL three-neck round-bottom flask. The reaction mixture was refluxed for 8 h at 118 °C. The product was washed with toluene to a neutral pH of the aqueous layer and kept for 12 h above anhydrous sodium sulfate; then, the solvent was distilled off to give 4.2 g of the product (yield 94%). ^1^H NMR (250 MHz, CDCl_3_) δ, ppm: 6.30–5.60 (m, 3H), 0.51–−0.30 (m, 9H). Molecular weight characteristics are shown in Table 1.

### 2.4. Synthesis of PDMS-75-Vin (MeVin/Me_2_ = 3/1) by Simultaneous Mixing

Dimethyldiethoxysilane (1.75 g, 0.012 mol), methylvinyldimethoxysilane (5.25 g, 0.040 mol), and acetic acid (28.6 mL, 0.5 mol) were added simultaneously to a 100 mL three-neck round-bottom flask. The reaction mixture was refluxed for 8 h at 118 °C. The product was washed with toluene to a neutral pH of the aqueous layer and kept for 12 h above anhydrous sodium sulfate; then, the solvent was distilled off to give 4.1 g of the product (yield 92%). ^1^H NMR (250 MHz, CDCl_3_) δ, ppm: 6.32–5.59 (m, 3H), 0.50–−0.29 (m, 5H). Molecular weight characteristics are shown in Table 1.

### 2.5. Synthesis of PDMS-25-Vin (MeVin/Me_2_ = 1/3) by Simultaneous Mixing

Dimethyldiethoxysilane (5.9 g, 0.040 mol), methylvinyldimethoxysilane (1.35 g, 0.012 mol), and acetic acid (28.6 mL, 0.5 mol) were added simultaneously to a 100 mL three-neck round-bottom flask. The reaction mixture was refluxed for 8 h at 118 °C. The product was washed with toluene to a neutral pH of the aqueous layer and kept for 12 h above anhydrous sodium sulfate; then, the solvent was distilled off to give 3.9 g of the product (yield 92%). ^1^H NMR (250 MHz, CDCl_3_) δ, ppm: 6.20–5.62 (m, 3H), 0.51–−0.21 (m, 21H). Molecular weight characteristics are shown in Table 1.

### 2.6. Synthesis of PDMS-100-Vin (MeVin/Me_2_ = 1/0) by Simultaneous Mixing

Methylvinyldimethoxysilane (7.5 g, 0.057 mol) and acetic acid (35.9 mL, 0.57 mol) were added simultaneously to a 100 mL three-neck round-bottom flask. The reaction mixture was refluxed for 8 h at 118 °C. The product was washed with toluene to a neutral pH of the aqueous layer and kept for 12 h above anhydrous sodium sulfate; then, the solvent was distilled off to give 5.3 g of the product (yield 90%). ^1^H NMR (250 MHz, CDCl_3_) δ, ppm: 6.20–5.63 (m, 3H), 0.49–−0.15 (m, 3H). Molecular weight characteristics are shown in Table 1.

### 2.7. Synthesis of PDMS-50-Vin (MeVin/Me_2_ = 1/1) by Slow Injection

Half of the acetic acid (14.3 mL, 0.25 mol) was added to a 100 mL three-neck round-bottom flask. Then, dimethyldiethoxysilane (3.7 g, 0.025 mol) and methylvinyldimethoxysilane (3.3 g, 0.025 mol) in the second half of the acetic acid (14.3 mL, 0.25 mol) were added into the system in a controlled manner using a dosing syringe pump at a rate of 0.1 mL/min. Then, the reaction mixture was refluxed for 6 h at 118 °C until complete alkoxy groups conversion (time after full addition of components). The product was washed with toluene to a neutral pH of the aqueous layer and kept for 12 h above anhydrous sodium sulfate; then, the solvent was distilled off to give 4.1 g of the product (yield 93%). ^1^H NMR (250 MHz, CDCl_3_) δ, ppm: 6.40–5.55 (m, 3H), 0.13 (dddd, J = 17.3, 7.4, 5.5, 2.2 Hz, 9H). Molecular weight characteristics are shown in Table 1.

### 2.8. Synthesis of PDMS-75-Vin (MeVin/Me_2_ = 3/1) by Slow Injection

Half of the acetic acid (14.3 mL, 0.25 mol) was added to a 100 mL three-neck round-bottom flask. Then, dimethyldiethoxysilane (1.75 g, 0.012 mol) and methylvinyldimethoxysilane (5.25 g, 0.040 mol) in the second half of the acetic acid (14.3 mL, 0.25 mol) were added into the system in a controlled manner using a dosing syringe pump at a rate of 0.1 mL/min. Then, the reaction mixture was refluxed for 6 h at 118 °C until complete alkoxy groups conversion (time after full addition of components).The product was washed with toluene to a neutral pH of the aqueous layer and kept for 12 h above anhydrous sodium sulfate; then, the solvent was distilled off to give 3.9 g of the product (yield 89%).^1^H NMR (250 MHz, CDCl_3_) δ, ppm: 6.29–5.51 (m, 3H), 0.60–−0.39 (m, 5H). Molecular weight characteristics are shown in Table 1.

### 2.9. Synthesis of PDMS-25-Vin (MeVin/Me_2_ = 1/3) by Slow Injection

Half of the acetic acid (14.3 mL, 0.25 mol) was added to a 100 mL three-neck round-bottom flask. Then, dimethyldiethoxysilane (5.9 g, 0.040 mol) and methylvinyldimethoxysilane (1.35 g, 0.012 mol) in the second half of the acetic acid (14.3 mL, 0.25 mol) were added into the system in a controlled manner using a dosing syringe pump at a rate of 0.1 mL/min. Then, the reaction mixture was refluxed for 6 h at 118 °C until complete alkoxy groups conversion (time after full addition of components). The product was washed with toluene to a neutral pH of the aqueous layer and kept for 12 h above anhydrous sodium sulfate; then, the solvent was distilled off to give 4.4 g of the product (yield 90%).^1^H NMR (250 MHz, CDCl_3_) δ, ppm: 6.33–5.46 (m, 3H), 0.68–−0.36 (m, 21H). Molecular weight characteristics are shown in Table 1.

### 2.10. Synthesis of PDMS-100-Vin (MeVin/Me_2_ = 1/0) by Slow Injection

Half of the acetic acid (16.3 mL, 0.28 mol) was added to a 100 mL three-neck round-bottom flask. Then, methylvinyldimethoxysilane (7.5 g, 0.057 mol) in the second half of the acetic acid (16.3 mL, 0.28 mol) was added into the system in a controlled manner using a dosing syringe pump at a rate of 0.1 mL/min. The reaction mixture was refluxed for 6 h at 118 °C. Then, the reaction mixture was refluxed for 6 h at 118 °C until complete alkoxy groups conversion (time after full addition of components). The product was washed with toluene to a neutral pH of the aqueous layer and kept for 12 h above anhydrous sodium sulfate; then, the solvent was distilled off to give 5.4 g of the product (yield 91%). ^1^H NMR (250 MHz, CDCl_3_) δ, ppm: 6.30–5.61 (m, 3H), 0.51–−0.29 (m, 3H). Molecular weight characteristics are shown in Table 1.

### 2.11. Blocking of Condensation Products

A 10 wt. % solution of PDMS-X-Vin oligomer (1.0 g, 0.01 mol) in toluene, chlorotrimethylsilane (2.67 g, 0.025 mol), and pyridine (1.93 g, 0.025 mol) was added to a 20 mL round-bottom flask. The reaction mixture was stirred for 2 h. The product was washed to a neutral pH of the aqueous layer and kept for 12 h above anhydrous sodium sulfate; then, the solvent was distilled off. ^1^H NMR (250 MHz, CDCl_3_) δ, ppm: 6.23–5.58 (m, 1H), 0.47–−0.26 (m, 3H). IR 2954 cm^−1^ ν_as_ (CH_3_-), 2921 cm^−1^ ν_as_ (-CH_2_-), 1465 cm^−1^ ν_um_ (-CH_2_-), and 1259 cm^−1^ ν (Si-CH_3_). GC–MS (EI, 70 eV) spectrum of the low-molecular-weight fraction of the blocked product: 293.15 [Si_4_O_4_C_8_H_21_]^+^; 305.15 [Si_4_O_4_C_9_H_21_]^+^; 317.15 [Si_4_O_4_C_10_H_21_]^+^; 329.15 [Si_4_O_4_C_11_H_21_]^+^; 391.2 [Si_5_O_5_C_12_H_27_]^+^; 403.2 [Si_5_O_5_C_13_H_27_]^+^; 415.2 [Si_5_O_5_C_14_H_27_]^+^; 477.25 [Si_6_O_6_C_15_H_33_]^+^; 489.2 [Si_6_O_6_C_16_H_33_]^+^; and 501.2 [Si_6_O_6_C_17_H_33_]^+^.

### 2.12. Synthesis of PDMS-50-Vin (MeVin/Me_2_ = 1/1) by Slow Introduction with Rate of 0.3 mL/min

Half of the acetic acid (103 mL, 1.8 mol) was added to a 500 mL three-neck round-bottom flask. Then, dimethyldiethoxysilane (26.7 g, 0.18 mol) and methylvinyldimethoxysilane (23.8 g, 0.18 mol) in the second half of the acetic acid (103 mL, 1.8 mol) were added into the system in a controlled manner using a dropping funnel at a rate of 0.3 mL/min. The reaction mixture was refluxed for 6 h at 118 °C. The product was washed with MTBE to a neutral pH of the aqueous layer and kept for 12 h above anhydrous sodium sulfate; then, the solvent was distilled off. ^1^H NMR (250 MHz, CDCl_3_) δ, ppm 6.49–5.42 (m, 3H), 0.55–−0.39 (m, 9H). Mp = 1200, Mn = 950, Mw = 1300, and PDI = 1.4 by GPC. The impact of rate on the composition of cyclic and linear products are shown in Table 2.

### 2.13. Synthesis of PDMS-75-Vin (MeVin/Me_2_ = 3/1) by Slow Introduction with Rate of 0.3 mL/min

Half of the acetic acid (103 mL, 1.8 mol) was added to a 500 mL three-neck round-bottom flask. Then, dimethyldiethoxysilane (15.4 g, 0.09 mol) and methylvinyldimethoxysilane (40.4 g, 0.27 mol) in the second half of the acetic acid (103 mL, 1.8 mol) were added into the system in a controlled manner using a dropping funnel at a rate of 0.3 mL/min. The reaction mixture was refluxed for 6 h at 118 °C. The product was washed with MTBE to a neutral pH of the aqueous layer and kept for 12 h above anhydrous sodium sulfate; then, the solvent was distilled off. ^1^H NMR (250 MHz, CDCl_3_) δ, ppm: 6.46–5.36 (m, 3H), 0.13 (dtd, J = 16.7, 5.9, 3.0 Hz, 5H). Mp = 1000, Mn = 850, Mw = 1100, and PDI = 1.3 by GPC. The impact of rate on the composition of cyclic and linear products are shown in Table 2.

### 2.14. Synthesis of PDMS-25-Vin (MeVin/Me_2_ = 1/3) by Slow Introduction with Rate of 0.3 mL/min

Half of the acetic acid (103 mL, 1.8 mol) was added to a 500 mL three-neck round-bottom flask. Then, dimethyldiethoxysilane (46.3 g, 0.27 mol) and methylvinyldimethoxysilane (13.5 g, 0.09 mol) in the second half of the acetic acid (103 mL, 1.8 mol) were added into the system in a controlled manner using a dropping funnel at a rate of 0.3 mL/min. The reaction mixture was refluxed for 6 h at 118 °C. The product was washed with MTBE to a neutral pH of the aqueous layer and kept for 12 h above anhydrous sodium sulfate; then, the solvent was distilled off. ^1^H NMR (250 MHz, CDCl_3_) δ, ppm: 6.35–5.53 (m, 3H), 0.57–−0.28 (m, 21H). Mp = 1050, Mn = 900, Mw = 1200, and PDI = 1.3 by GPC. The impact of rate on the composition of cyclic and linear products are shown in Table 2.

### 2.15. Oligo(methylvinyl)(dimethyl)siloxane Thermal Condensation

The product of polycondensation in acetic acid was added to a 500 mL round-bottom flask. Then, acetic acid was removed at low pressure in a rotary evaporator. The resulting mixture was poured into a 100 mL round-bottom flask and stirred in a rotary evaporator for 5 h at various temperatures (50–150 °C) in vacuo (1 torr). ^1^H NMR (250 MHz, CDCl_3_) δ, ppm: 6.26–5.56 (m, 3H), 0.51–−0.43 (m, 9H). Molecular weight characteristics are shown in Table 3, Table 4 and Table 5.

### 2.16. Oligo(methylvinyl)(dimethyl)siloxane Thermal Condensation with AcOK

The product of polycondensation in acetic acid, AcOK (1 wt. %), was added to a 500 mL round-bottom flask. Then, the mixture was stirred for 5 h at 150 °C in vacuo (1 torr). ^1^H NMR (250 MHz, CDCl_3_) δ, ppm: 6.36–5.38 (m, 3H), 0.62–−0.38 (m, 9H). Molecular weight characteristics are shown in Table 3, Table 4 and Table 5.

### 2.17. The Analysis of the Microstructure of Copolymers

The microstructure of copolymers was determined using parameter R “degree of randomness” [39,40], according to the following formula:

R = (DVD + VDV)/(2DDD + DVD + VDV) + (DVD + VDV)/(2VVV + DVD + VDV)

where VDV, VDD, DDD, DVD, VVD, and VVV are integral intensities of the triad sequence from the ^29^Si NMR spectra.

At R > 1, polymer has a tendency toward the alternation of units, and at R = 2, it is an entirely alternating copolymer. At R < 1, polymer has a block structure, and at R = 0, it is completely a block copolymer or a mixture of two homopolymers. The value R = 1 indicates a random distribution of units in the copolymer.

## 3. Results and Discussion

### 3.1. Copolycondensation of Dimethyldiethoxysilane and Methylvinyldimethoxysilane in Active Medium

Commercially available methylvinyldimethoxy- MeVinSi(OMe)_2_ and dimethyldiethoxysilane Me_2_Si(OEt)_2_ served as initial reagents to synthesize vinyl-containing polydimethylsiloxanes. Previous research has shown that the polycondensation process of alkoxysilanes in an active medium proceeds through a series of steps: the acidolysis of alkoxy groups, esterification involving free alcohol and acetic acid, the hydrolysis of acetoxy derivative from acidolysis with water, and the heterofunctional polycondensation of acetoxy- and hydroxy-derivatives, leading to the siloxane bond formation. Since the siloxane bond formation involves acetoxysilane rather than alkoxysilane, the type of alkoxy group does not impact the composition of the resulting product [34].

**Figure 1:**
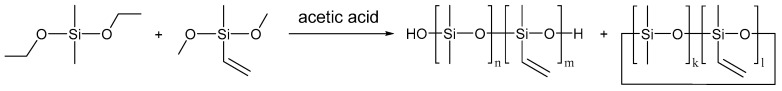
Scheme of copolycondensation of Me_2_Si(OEt)_2_ and MeVinSi(OMe)_2_ in active medium.

Experiments conducted with Me_2_Si(OEt)_2_ have previously highlighted the pivotal influence of the reagent introduction method on the prevalent formation of linear or cyclic products [35]. Therefore, this study aimed to evaluate the influence of reagent addition order on the composition of the resulting products and molecular weights across various molar ratios of Me_2_Si(OEt)_2_ and MeVinSi(OMe)_2_ (0/100, 25/75, 50/50, 75/25). The general scheme of its copolycondensation in an excess of anhydrous acetic acid is presented in Figure 1.

**Figure 2:**
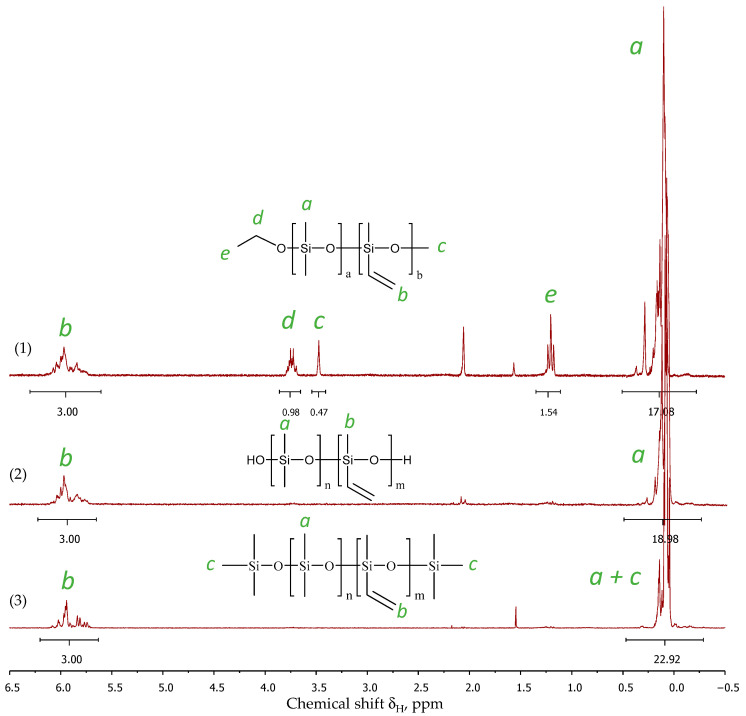
^1^H NMR spectra of PDMS-25-Vin (vacuumized samples) after 2 h of reaction (**1**), after 8 h of reaction (**2**), and blocked product (**3**). The letters a-e indicate the signals of protons of the corresponding groups.

The process was monitored using ^1^H NMR spectroscopy with the disappearance of the proton signals of the alkoxy groups. Figure 2 shows the ^1^H NMR spectra of the dimethyldiethoxy- and methylvinyldimethoxysilane copolycondensation products in a molar ratio of 75/25 (PDMS-25-Vin) after 2 and 8 h (Figure 2 (1) and (2), respectively). The signals between δ = 0.10 and δ = 0.37 ppm are assigned to the methyl groups, and the signals in the range of 5.77–6.07 ppm are attributed to the vinyl groups. The disappearance of the methoxy group at 3.5 ppm of MeVinSi(OMe)_2_ and the ethoxy group at 3.7 ppm (CH_3_-) and 1.2 ppm (-CH_2_-) of Me_2_Si(OEt)_2_ clearly indicate the reaction’s completion. At the reaction temperature of 120 °C, the alkoxy groups were fully converted within 6–8 h (Table 1).

**Figure 3:**
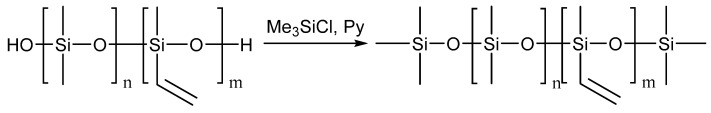
General scheme of ologo(methylvinyl)(dimethyl)siloxanes trimethylsilylation.

To stabilize the composition and prevent further condensation of the hydroxyl groups, the siloxane products were blocked with trimethyl chlorosilane, as described in [32] (Figure 3).

The efficiency of blocking was assessed by the absence of the absorption band at 3500 cm^−1^ on the IR spectrum of the product after blocking. IR spectra of the copolycondensation products derived from Me_2_Si(OEt)_2_ and MeVinSi(OMe)_2_ are presented in Figure S1 (see the Supplementary Materials).

**Figure 4:**
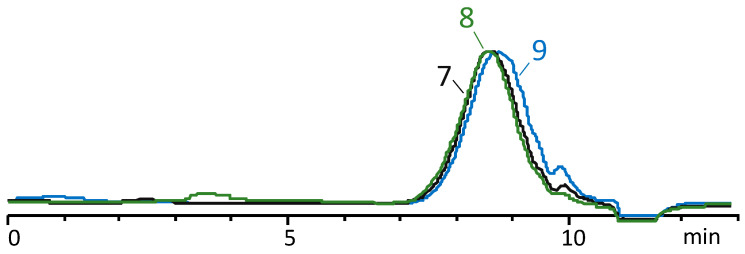
GPC curve of the blocked product. The labels 7, 8, 9 correspond to the numbers in the Table 1.

**Figure 5:**
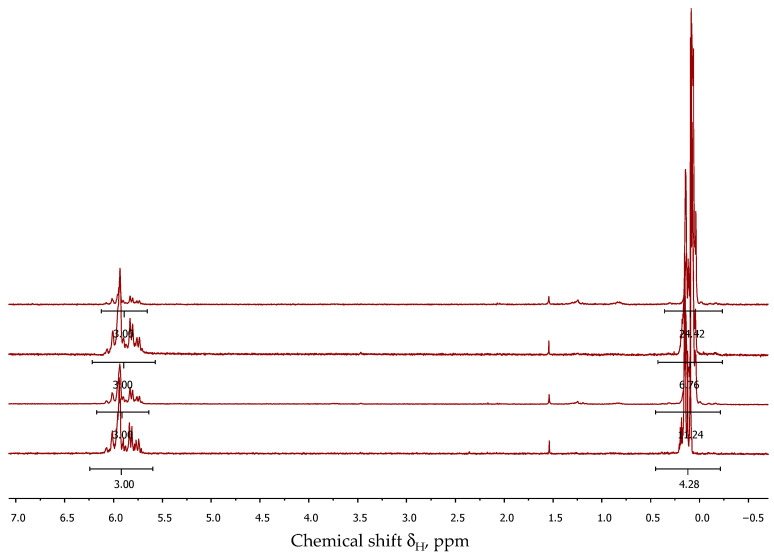
^1^H NMR spectra of the cooligomers after trimethylsilylation.

Following trimethylsilylation, the product was distilled, and the volatile components were analyzed via GLC and GC-MS to determine the composition (Figure S2, Table S1). Non-volatile components were evaluated using GPCs to assess the molecular weights of the linear products (Figure 4). Additionally, ^1^H NMR spectroscopy was used to determine the ratio of methylvinyl- and dimethylsiloxane units in the oligomers, as well as to determine the content of hydroxyl groups (Figure 5). The hydrolysis conditions, an overview of the chemical composition of copolymers, and the number of OH groups, as well as the molecular weight and its distribution obtained through GPC measurements, are summarized in Table 1.

The identification of volatile products using GLC was difficult because of the presence of a wide array of cyclosiloxanes with varying compositions that have similar boiling points. As a result, the latter are poorly separated during the chromatography (see Figure S2 in Supplementary Materials). This problem was solved using the GC-MS method. Figure S3 (see Figure S3 in Supplementary Materials) exemplifies the GC-MS spectrum of the volatile condensation products. The composition of the volatile components determined using GC-MS is given in Table S1. These volatile products consist primarily of cyclic oligomers, predominantly in a copolymer composition with 3–7 siloxane units. The formation of homocyclic compounds reached up to 25% of the total number of cycles observed with a non-stoichiometric ratio of monomers (Table S2 № 2, 4, 6, 8), while with stoichiometric ratios of monomers, homocycles comprise less than 5% of the total volatile products (Table S2, № 3, 7). The correlation between dimethyl- and methylvinyl units in mixed-structure cycles coincides with the monomer ratio, even in cases with non-stoichiometric ratios, resulting in mixed cycles enriched with an excess of specific monomer units. Moreover, the ratio of dimethyl- and methylvinyl units in cycles of mixed structure correlates with the ratio of monomers. For instance, in a 25/75 monomer ratio, cycles D_3_V_1_, D_2_V_2_, D_5_V_1_, D_4_V_2_, D_4_V_1_, and D_3_V_2_ constitute 68% of the total volatile products, while 25% represent a mixture from D_3_ to D_7_, and 7% are mixed cycles enriched in methylvinyl units. Similarly, with a 75/25 monomer ratio, cycles D_1_V_2_, D_2_V_2_, D_1_V_3_, and D_2_V_3_ make up 69% of the total volatile products, with 28.5% comprising a mixture from V_3_ to V_7_, and 2.5% are mixed cycles enriched in dimethyl units.

The composition of non-volatile products, regardless of polycondensation conditions, corresponded to the calculated monomer ratio. This was determined by the ratio of the integral intensities of protons of methyl and vinyl groups in the regions 0.10–0.37 ppm and 5.77–6.07 ppm, respectively, for vacuumed unblocked product (Table 1).

Figure 4 shows the GPC curves of non-volatile products. According to the data given in Table 1, all non-volatile products are characterized by a narrow molecular weight distribution, M_w_/M_n_ is 1.3–1.4, and a low molecular weight value between 1300 and 900.

Analysis of the data in Table 1 indicates that, akin to the polycondensation of Me_2_Si(OEt)_2_ in an active medium [35], the sequence of reagent addition influences the ratio of cyclic and linear products. In the case of the polycondensation of MeVinSi(OMe)_2_ under simultaneous mixing conditions, a larger number of linear oligomers are formed compared to Me_2_Si(OEt)_2_—58% and 45%, respectively (Table 1 № 1, 5). Shifting to slow injection of the monomer into the reaction mixture increased the yield of linear oligomethylvinylsiloxanes to 73% (Table 1 № 6). A similar trend was observed for all variants of Me_2_Si(OEt)_2_ and MeVinSi(OMe)_2_ mixtures. The order of reagent introduction does not significantly alter the composition of copolymer cyclic and linear siloxanes; however, it slightly impacts the size and molecular weight of oligomers. Oligomers obtained by slow injection are characterized by a higher molecular weight of 1400–1700 compared to 1150–1000 in the case of simultaneous mixing (Table 1 № 1–4, 6–9). This can be explained by the higher contribution of the heterofunctional condensation of acetoxy- and hydroxy-derivatives of monomers in forming the siloxane bond in the case of slow injection. However, this effect is evident only for a slow introduction rate of 0.1 mL/min. An increase to 0.3 mL/min diminishes this effect, leading to a ratio of cyclic and linear products similar to those obtained under simultaneous mixing (Table 2).

Thus, the use of the slow injection of monomers has prospects only for small quantities, whereas for scaling up operations, simultaneous mixing proves to be more advantageous.

In summary, the copolycondensation of Me_2_Si(OEt)_2_ and MeVinSi(OMe)_2_ in an active medium demonstrates the potential for obtaining α,ω-dihydroxyoligo(methylvinyl)(dimethyl)siloxanes with a targeted unit ratio, with yields between 60 and 80% contingent on the reaction conditions.

### 3.2. Enhancing the Molecular Weight of Oligodimethylmethylvinylsiloxanes

**Figure 6:**
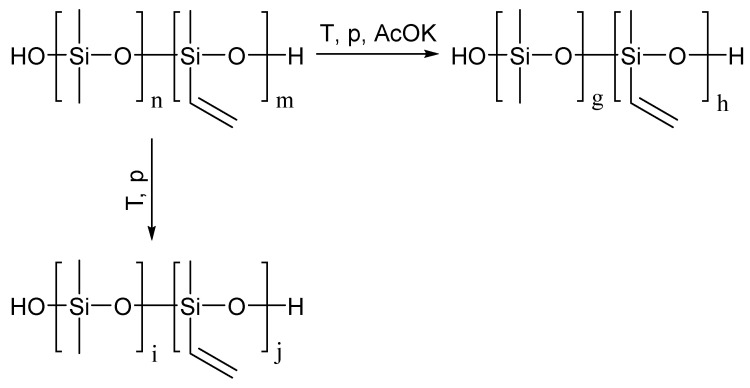
Scheme of thermal condensation in vacuum.

The polycondensation of bifunctional alkoxysilanes in an active medium leads to the formation of low-molecular-weight oligomeric products, as reported in [32]. However, the elevation of their molecular weight can be achieved through subsequent thermal condensation (Figure 6).

It is possible to increase the molecular weight of dimethylsiloxane oligomers up to 70,000 via thermal condensation at 50–150 °C/1 mmHg. For oligomers containing phenyl and benzyl substituents at the silicon atom, this effect requires the presence of a catalyst—potassium acetate. Its utilization increases the molecular weight without depolymerization processes [36,37], commonly observed when strong acids and alkalis are used. The approach to enhance the molecular weight of the oligosiloxanes in this study replicated the above-mentioned methods. Sequential exposure for 5 h at temperatures of 50, 100, and 150 °C in vacuum was used for samples after the simultaneous mixing method. The monitoring of the process involved tracking changes in the molecular weight and cyclic product content using the GPCs.

**Figure 7:**
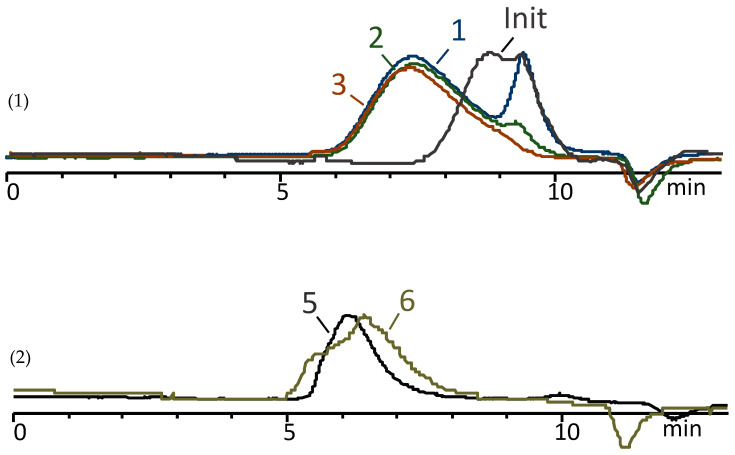
GPC curves of the products after thermal condensation in vacuum, where (**1**) GPC curves using Phenomenex column with a pore size 500 Å and (**2**) GPC curves using Phenomenex column with a pore size 10^4^ Å. The labels 1–6 correspond to the numbers in the Table 3.

For PDMS-100-Vin, raising the temperature to 50 °C led to a moderate increase in M_W_ from 1100 to 4200 (Table 3, Figure 7 (1)).

**Figure 8:**
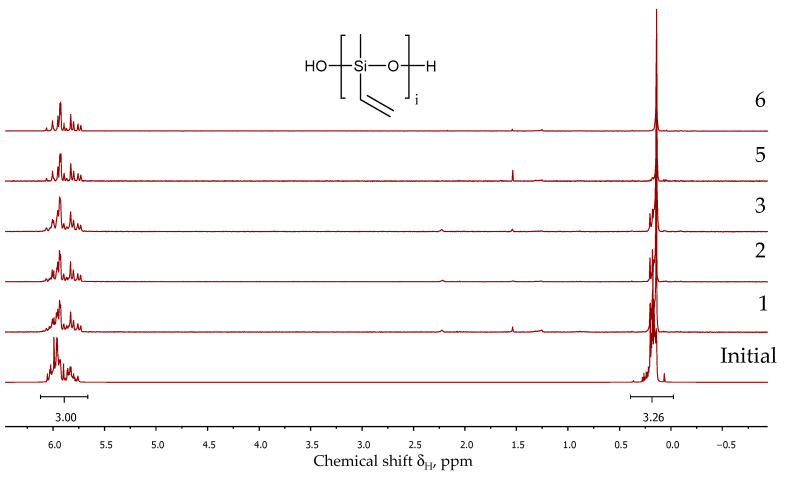
^1^H NMR spectra of the products after thermal condensation in vacuum. The labels 1–6 correspond to the numbers in the Table 3.

Further elevation of the temperature up to 150 °C did not affect the molecular mass. Despite the temperature, the ratio of methyl and vinyl units determined by ^1^H NMR spectroscopy matched the calculated values. This stability suggests the resilience of thermally labile vinyl groups under the examined conditions (Figure 8). However, introducing AcOK into the system after extended heat treatment induced gel formation.

Meanwhile, the thermal condensation of PDMS-100-Vin in the presence of AcOK immediately after the polycondensation of MeVinSi(OMe)_2_ allows for an increase in the MM to 29,000 after 5 h (Table 3 № 5, Figure 7 (2)). The subsequent isolation and heat treatment of the product in the presence of fresh AcOK boosted up the molecular weight to 124,000 (Table 3, № 6, Figure 7 (2)) with the complete preservation of vinyl groups. Notably, during the thermal condensation, low-molecular-weight compounds were distilled, and the copolymers generated under such conditions were devoid of cyclic products.

We also studied the thermal condensation process of oligomers with a different ratio of dimethyl- and methylvinylsiloxyl compounds derived from the copolycondensation of Me_2_Si(OEt)_2_ and MeVinSi(OMe)_2_ in an active medium. The results are outlined in Table 4.

Unlike oligomethylvinylsiloxane oligomers, the introduction of dimethyl units significantly increases the MM of copolymers even at 50 °C—the observed values of the MM for oligomers escalated from 1500 to 8000 (Table 4 № 1), reaching up to 20,000 with subsequent temperature elevation to 100–150 °C (Table 4 № 2, 3).

Using AcOK as a catalyst for the thermal condensation of cooligomers at 150 °C yielded similar molecular weights (ca. 20,000) only within 5 h (Table 4 № 4). According to the ^1^H NMR spectroscopy data, the ratio of dimethylsiloxy and methylvinylsiloxy links remained constant for all oligomers and over the entire range of thermal condensation conditions.

Thus, it was shown that the copolycondensation of dimethyl and methyl vinyl dialkoxysilanes in an active medium makes it possible to obtain vinyl-containing dimethylsiloxane oligomers and polymers with controlled content of vinyl group condensation and molecular weight characteristics. It is possible to implement the second stage without cyclization:

- Only in the presence of a catalyst for the condensation of silanol groups—AcOK in the case of methylvinylsiloxane oligomers PDMS-100-Vin;
- Both in the presence of a catalyst and without it in the of case oligomers with a mixed composition. Simultaneously with postcondensation, the cycles formed at the first stage of polycondensation in the active medium are distilled off, which makes it possible to obtain a final polymer that does not contain low-molecular-weight cyclic impurities (according to GPCs).

It should be noted that this division of the process into two stages is quite arbitrary, and it is possible to implement the entire two-stage process in one-pot mode, which significantly distinguishes it from traditional approaches based on the catalytic hydrolytic polycondensation of functional silanes, accompanied by intermediate stages of neutralization, filtration, and washing of product and the stage of purifying the final polymer from cycles.

### 3.3. The Study of Polymer Microstructure

**Figure 9:**
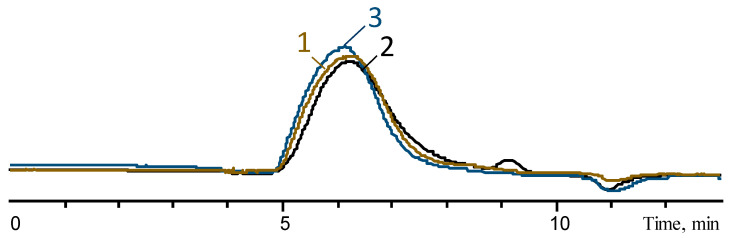
GPC curves of PDMS-X-Vin after thermal condensation. The labels 1–3 correspond to the numbers in the Table 5.

To study the microstructure of copolymers, a series of samples was synthesized. A series of copolymers with comparable molecular weights was obtained by the copolycondensation of Me_2_Si(OEt)_2_ and MeVinSi(OMe)_2_ in an active medium followed by thermal condensation at 150 °C at 1 mmHg (Table 5, Figure 9). Synthesized vinyl functional PDMSs are characterized by a monomodal molecular weight distribution and a polydispersity coefficient ranging from 1.5 to 1.9.

**Figure 10:**
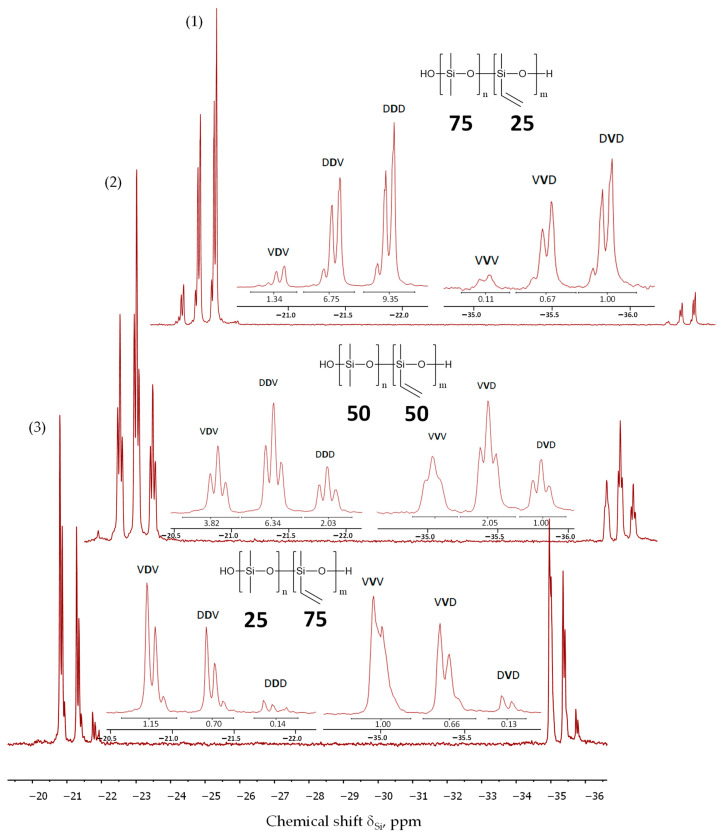
^29^Si NMR spectrum of the copolymers. The labels (1)–(3) correspond to the numbers in the Table 5.

Their microstructure was examined with ^29^Si NMR spectroscopy, and the signals of structural fragments were interpreted at the triad sequence level (Figure 10) [39]. Figure 10 shows the ^29^Si NMR spectra for PDMS-75-Vin, PDMS-50-Vin, and PDMS-25-Vin copolymers. In the ^29^Si NMR spectra of the obtained copolymers, signals are observed in the range from −34.9 to –36.0 ppm, corresponding to the V-units, and in the range from –20.7 to –22.1 ppm, belonging to the D-units of the obtained copolymer. The correlation of triads was carried out on the basis of data from the work [40]. In Table 6, all the studied copolymers are characterized by the presence of six types of triads, VDV, VDD, DDD, VVD, DVD, and VVV, where D = Me_2_SiO and V = MeVinSiO.

In the PDMS-25-Vin copolymer, the mole fraction of block V-centered VVV triads is only 1%, and as the content of methyl vinylsiloxane units increases, this fraction increases, and in the PDMS-50-Vin and PDMS-75-Vin copolymers, it is 7 and 26%, respectively. The PDMS-25-Vin synthesized in this work has a statistical distribution of units (R~1), while the copolymers PDMS-50-Vin and PDMS-75-Vin show a slight tendency to alternate (R~1.2) (Table 6).

## 4. Conclusions

The copolycondensation of Me_2_Si(OEt)_2_ and MeVinSi(OMe)_2_ in an active medium followed by thermal condensation in a vacuum resulted in copoly(methylvinyl)(dimethyl)siloxanes with controlled molecular weights (1500–20,000 a.m.) and the precise content of functional methylvinylsiloxane units. The division of the process into two stages is formal, since at the postcondensation stage the distillation of cycles occurs simultaneously, which makes it possible to obtain a final polymer free of cycles. In this case, the composition of the resulting vinyl-containing PDMS corresponds to the calculated ratio of monomers. The microstructure study of the copolymers synthesized revealed that the formation of the products was characterized by a random distribution of units (R~1) during copolymerization. However, an increase in the number of vinyl-containing monomers leads to an increase in the R parameter, thereby increasing the tendency for alternating links in the copolymer.

## Figures and Tables

**Table 1:** Composition and molecular weight characteristics of poly(methylvinyl)(dimethyl)siloxanes.

№	Process Conditions			Characteristics									
	PDMS-X-Vin	t ^a^, h	N ^b^ (HO), wt. %	Products’ Composition, wt. %				Molecular Weight Characteristics of L, wt. %				MeVinSiO/ Me_2_SiO for L	
				D_3–7_	D_n_V_m_	V_3–7_	L	M_p_	M_w_	M_n_	PDI	Th.	Real
1	PDMS-100-Vin-Ⅰ	8	7.3	-	-	42	58	1150	860	670	1.3	-	-
2	PDMS-25-Vin-Ⅰ	8	4.8	10	35.5	-	55	1000	1050	750	1.4	3/1	3/0.75
3	PDMS-50-Vin-Ⅰ	8	6.5	1	38.5	0.5	60	1000	870	660	1.3	1/1	1/0.85
4	PDMS-75-Vin-Ⅰ	8	6.8	-	34.6	2.4	63	1000	900	700	1.3	1/3	1/2.22
5	PDMS-0-Vin-Ⅰ **	-	-	55	-	-	45	1700	-	-	-	-	-
6	PDMS-100-Vin-Ⅱ	6 *	7.0	-	-	27	73	1600	900	680	1.3	-	-
7	PDMS-25-Vin-Ⅱ	6 *	4.7	7	23	-	70	1600	1200	900	1.3	3/1	3/0.88
8	PDMS-50-Vin-Ⅱ	6 *	5.7	1	19.6	0.4	79	1700	1300	900	1.4	1/1	1/0.85
9	PDMS-75-Vin-Ⅱ	6 *	6.5	0.2	16	9.8	74	1400	1100	800	1.3	1/3	1/2.36
10	PDMS-0-Vin-Ⅱ **	-	-	12	-	-	88	1700	-	-	-	-	-

PDMS-X-Vin, where X- content of methylvinyl units in the copolymer: 0, 25, 50, 75, 100. I or II—process conditions, where I indicates simultaneous mixing and II indicates slow injection. ^a^ Reaction time. ^b^ The number of OH groups in the oligo(methylvinyl)dimethylsiloxanes, calculated from ^1^H NMR spectroscopy of polycondensation blocked products. * Time after full introduction of components. ** Data according to the literature [35].

**Table 2:** The impact of reagent mixing methods on the composition of cyclic and linear products.

PDMS-X-Vin	Monomer Introduction Rate	Cyclic/Linear, %/%
PDMS-75-Vin	0.1 mL/min	26/74
	0.3 mL/min	38/62
	Simultaneous mixing	45.5/54.5
PDMS-50-Vin	0.1 mL/min	21/79
	0.3 mL/min	35/65
	Simultaneous mixing	40/60
PDMS-25-Vin	0.1 mL/min	30/70
	0.3 mL/min	36/64
	Simultaneous mixing	37/63

**Table 3:** Conditions and characteristics of homopolymer obtained by thermal condensation in vacuum.

№	Process Conditions		Characteristics				
	t, h	T, °C/AcOK	Before Condensation		After Condensation		
			M_p_	Cycles Content, %	M_p_	Cycles Content, %	PDI
initial	-	25/-	1100	36	-	-	
1	5	50/-	1100	36	4200	26	1.49
2	5	100/-	4200	26	4200	9	1.55
3	5	150/-	4200	9	4700	0	1.73
4	5	150/AcOK	4700	0	gel	-	-
5	5	150/AcOK	1100	36	29,000	0	-
6	5	150/AcOK	29,000	0	124,000	0	-

**Table 4:** Conditions and characteristics of copolymers obtained by thermal condensation in vacuum.

№	Conditions		Characteristics											
			PDMS-75-Vin				PDMS-50-Vin				PDMS-25-Vin			
	t, h	T, °C/ AcOK	Before		After		Before		After		Before		After	
			M_p_	C *, %	M_p_	C *, %	M_p_	C *, %	M_p_	C *, %	M_p_	C *, %	M_p_	C *, %
1	5	50/-	1400	27	7500	17	1700	20	7900	10	1600	25	8000	9
2	5	100/-	7500	17	18,000	5	7900	10	17,500	4	8000	9	15,000	2
3	5	150/-	18,000	5	20,000	0	17,500	4	18,000	0	15,000	2	18,000	0
4	5	150/AcOK	1400	27	25,000	0	1700	20	22,000	0	1600	25	21,500	0

* The content of cycles determined by GPCs.

**Table 5:** Molecular weight characteristics of PDMS-X-Vin.

№	PDMS-X-Vin	Molecular Weight Characteristics			
		M_p_	M_w_	M_n_	PDI
1	PDMS-25-Vin	18,000	31,500	16,700	1.9
2	PDMS-50-Vin	18,000	29,700	20,000	1.5
3	PDMS-75-Vin	20,000	25,500	15,200	1.7

**Table 6:** Chemical shifts and integrated intensities of the silicon atom in the ^29^Si spectra of the copolymers.

Triad	δ_Si_, ppm	Integral Intensity of Triad (%)		
		PDMS-75-Vin (R = 1.21)	PDMS-50-Vin (R = 1.22)	PDMS-25-Vin (R = 1.02)
DVD	–(35.7–36.0)	3	6	5
DVV	–(35.3–35.6)	17	13	3
VVV	–(34.9–35.2)	26	7	1
DDD	–(21.7–22.1)	4	12	49
VDD	–(21.2–21.6)	19	39	35
VDV	–(20.7–21.1)	30	23	7

## Data Availability

Data available on request.

## Supplementary Materials

The following supporting information can be downloaded at https://www.mdpi.com/article/10.3390/polym16020257/s1, Figure S1: IR spectra of the blocking products; Figure S2: GLC of volatile products; Figure S3: GC-MS spectrum of volatile product PDMS-75-Vin: (a) general spectra, (b) at 4.920 min, (c) at 5.330 min, (d) at 5.723 min, (e) at 6.097 min, (f) at 6.463 min, (g) at 6.797 min, (h) at 7.113 min, and (i) at 7.493 min; Table S1: Characteristics of cyclosiloxanes; Table S2: Detailed composition of cycles.
