# Supplementary material for: Synthesis of Vinyl-Containing Polydimethylsiloxane in An Active Medium
Source: Polymers (Basel). 2024 Jan 16;16(2):257. doi: 10.3390/polym16020257 (PMC10819907; doi:10.3390/polym16020257)
Supplement: Supplementary file 1 [file polymers-16-00257-s001.zip › polymers-2792924-supplementary.pdf]

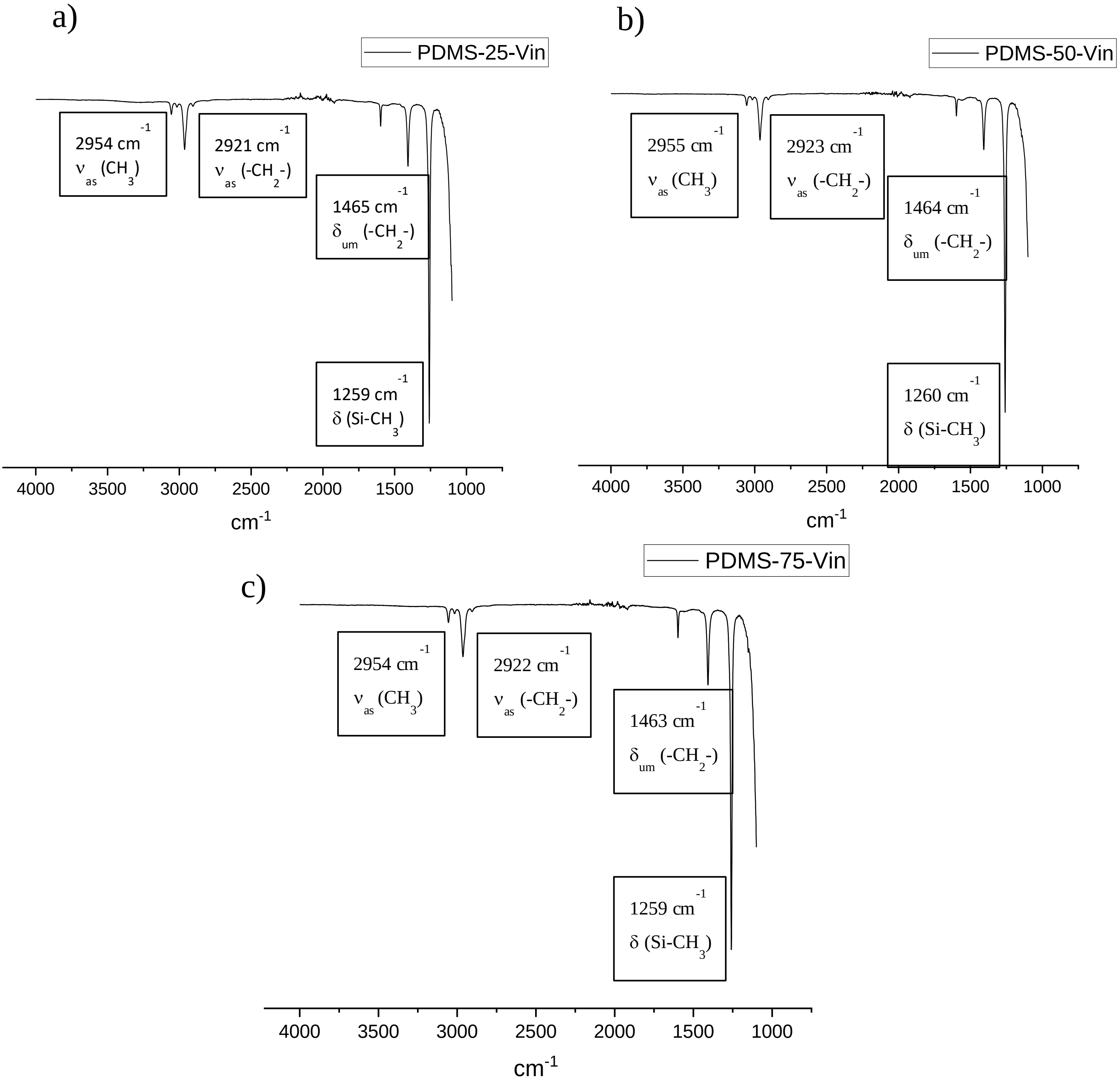

Figure S1. *IR spectra of the blocking products*

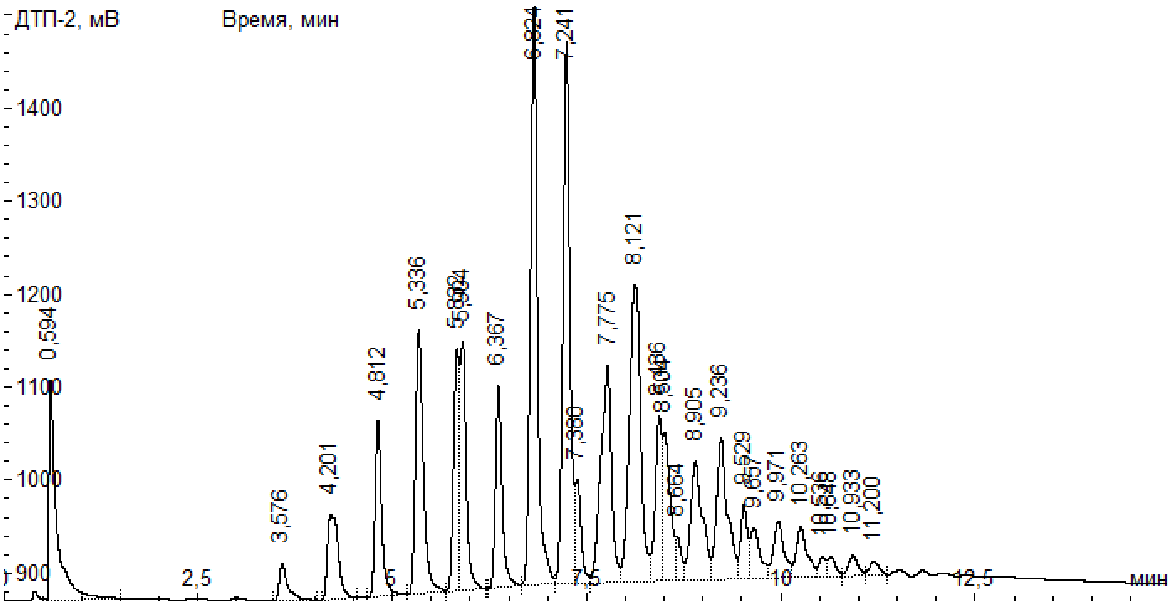

Figure S2. *GLC of volatile products*

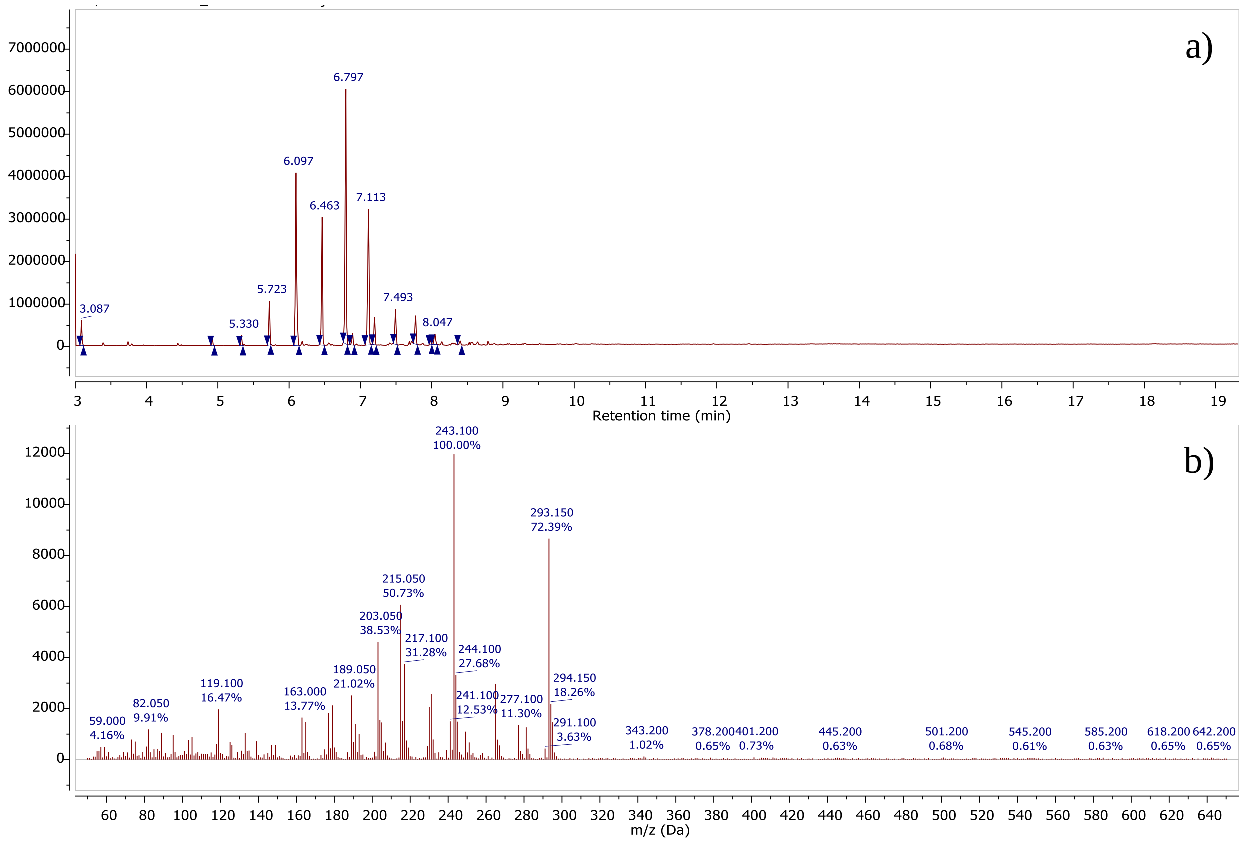

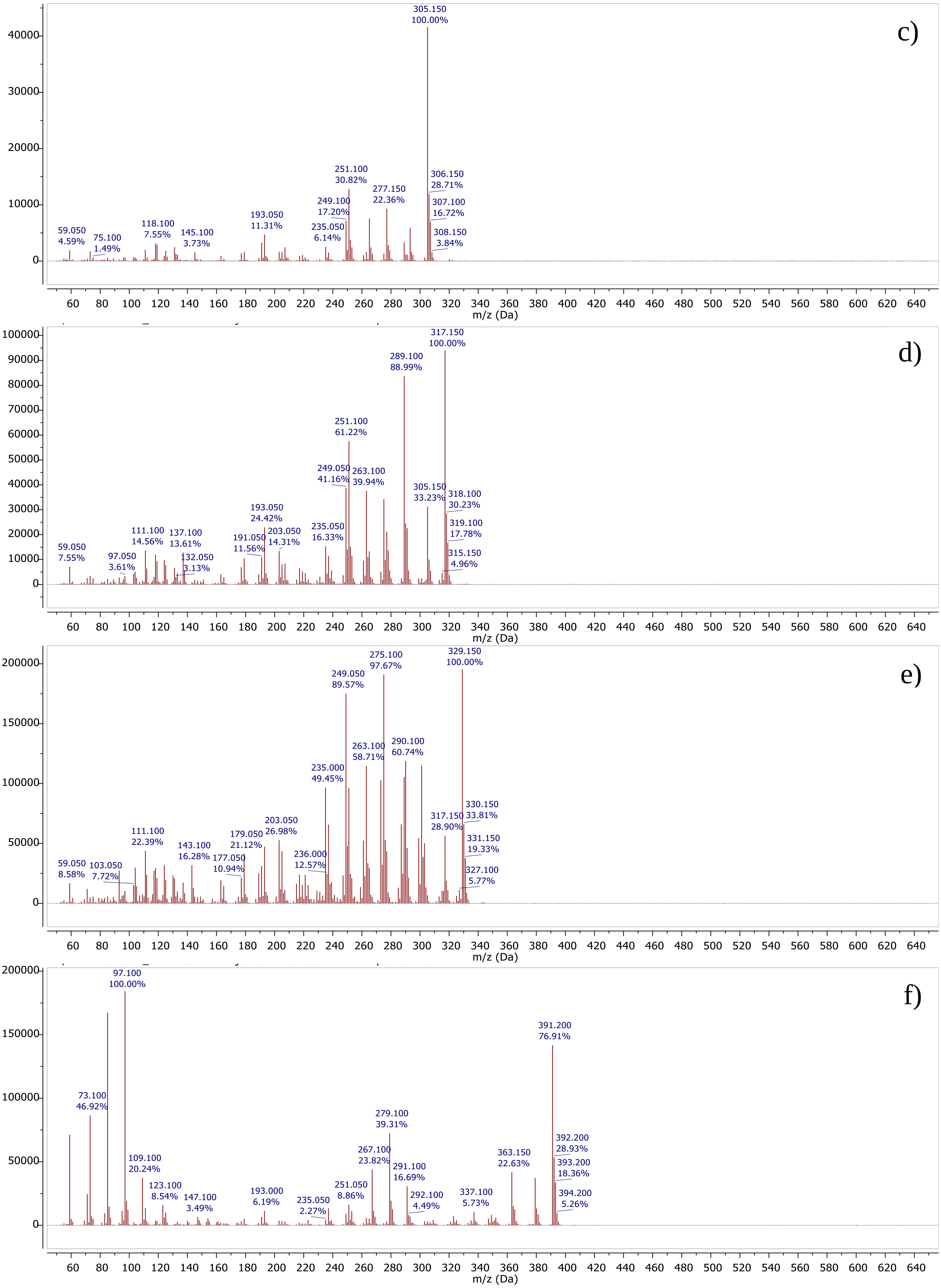
c)
305.150 100.00%
251.100 30.82%
277.150 22.36%
306.150 28.71%
307.100 16.72%
308.150 3.84%
249.100 17.20%
235.050 6.14%
193.050 11.31%
145.100 3.73%
118.100 7.55%
75.100 1.49%
59.050 4.59%
d)
317.150 100.00%
289.100 88.99%
251.100 61.22%
249.050 41.16%
263.100 39.94%
305.150 33.23%
318.100 30.23%
319.100 17.78%
315.150 4.96%
235.050 16.33%
193.050 24.42%
203.050 14.31%
191.050 11.56%
137.100 13.61%
132.050 3.13%
111.100 14.56%
97.050 3.61%
59.050 7.55%
e)
329.150 100.00%
275.100 97.67%
249.050 89.57%
263.100 58.71%
290.100 60.74%
235.000 49.45%
330.150 33.81%
317.150 28.90%
331.150 19.33%
327.100 5.77%
236.000 12.57%
203.050 26.98%
179.050 21.12%
177.050 10.94%
143.100 16.28%
111.100 22.39%
103.050 7.72%
59.050 8.58%
f)
97.100 100.00%
391.200 76.91%
73.100 46.92%
279.100 39.31%
267.100 23.82%
291.100 16.69%
292.100 4.49%
392.200 28.93%
393.200 18.36%
394.200 5.26%
363.150 22.63%
337.100 5.73%
251.050 8.86%
235.050 2.27%
193.000 6.19%
147.100 3.49%
123.100 8.54%
109.100 20.24%
m/z (Da)

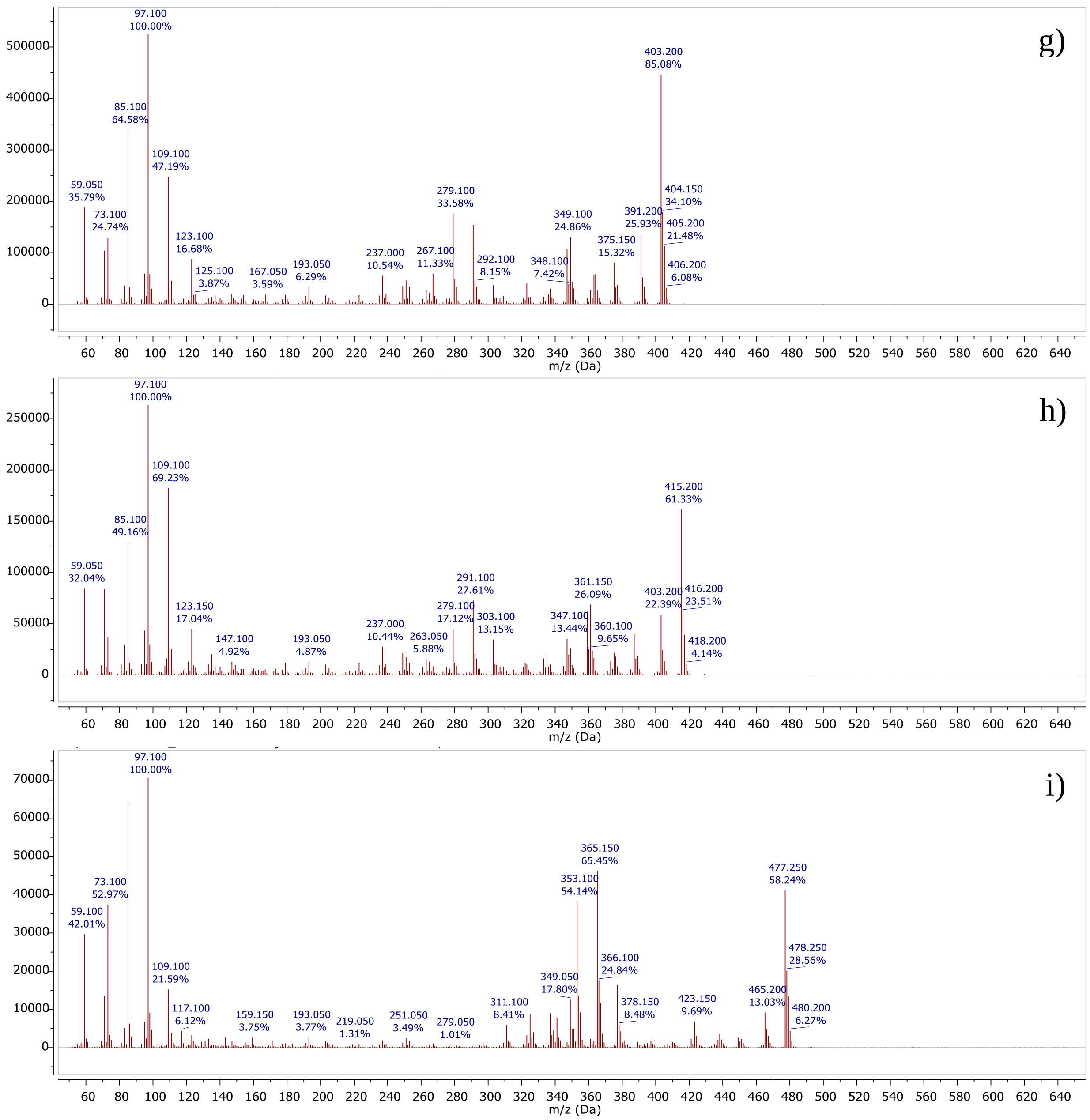

Figure S3. *GC-MS spectrum of volatile products PDMS-75-Vin: a) general spectra, b) at 4.920 min, c) at 5.330 min, d) at 5.723 min, e) at 6.097 min, f) at 6.463 min, g) at 6.797 min, h) at 7.113 min, i) at 7.493 min*

Table S1. *Characteristics of cyclosilcosanes*

№	m/z	Cycle	MM	Scheme	Boiling point, °C
1	219 $[Si_3O_3C_5H_{15}]^+$	D_3	234	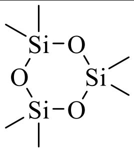	134
2	213 $[Si_3O_3C_6H_{15}]^+$	D_2V_1	234	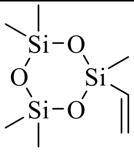	160

3	231 $[Si_3O_3C_7H_{15}]^+$	D_1V_2	246	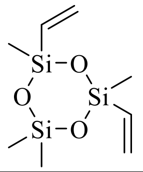	185.6±13
4	281 $[Si_4O_4C_7H_{21}]^+$	D_4	296	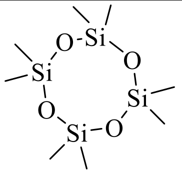	175
5	293 $[Si_4O_4C_8H_{21}]^+$	D_3V_1	308	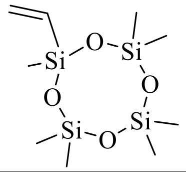	180
6	305 $[Si_4O_4C_9H_{21}]^+$	D_2V_2	320	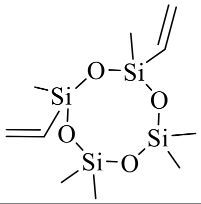	219.9±23
7	317 $[Si_4O_4C_{10}H_{21}]^+$	D_1V_3	332	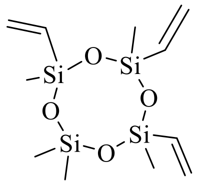	234±23
8	329 $[Si_4O_4C_{11}H_{21}]^+$	V_4	344	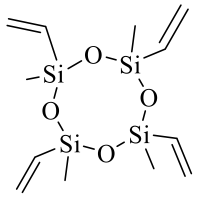	224
9	355 $[Si_5O_5C_9H_{27}]^+$	D_5	370	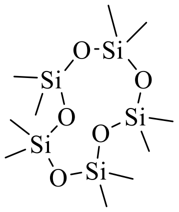	210
10	367 $[Si_5O_5C_{10}H_{27}]^+$	D_4V_1	382	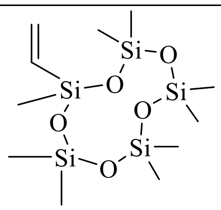	257.5±23
11	379 $[Si_5O_5C_{11}H_{27}]^+$	D_3V_2	394	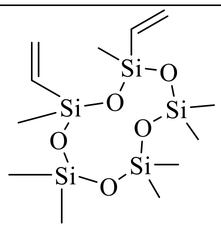	270.4±23

12	391<br>$[Si_5O_5C_{12}H_{27}]^+$	D_2V_3	406	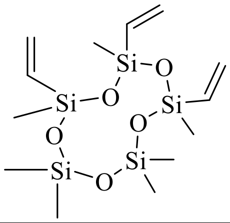	283,1±23
13	403<br>$[Si_5O_5C_{13}H_{27}]^+$	D_1V_4	418	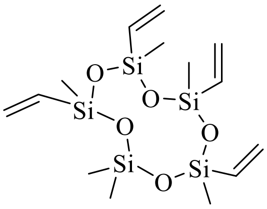	295.6±23
14	415<br>$[Si_5O_5C_{14}H_{27}]^+$	V_5	430	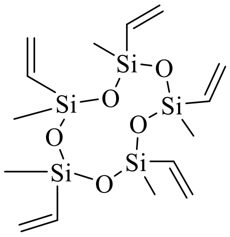	261-262
15	429<br>$[Si_6O_6C_{11}H_{33}]^+$	D_6	444	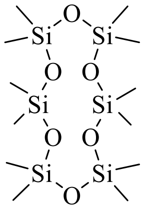	245
16	441<br>$[Si_6O_6C_{12}H_{33}]^+$	D_5V_1	456	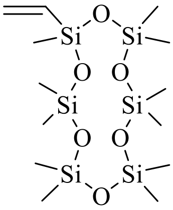	304.5±25
17	453<br>$[Si_6O_6C_{13}H_{33}]^+$	D_4V_2	468	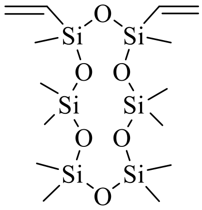	-
18	465<br>$[Si_6O_6C_{14}H_{33}]^+$	D_3V_3	480	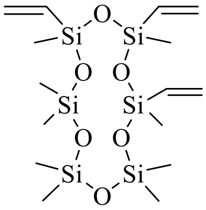	-
19	477<br>$[Si_6O_6C_{15}H_{33}]^+$	D_2V_4	492	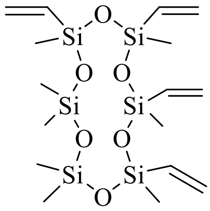	-

20	489 $[Si_6O_6C_{16}H_{33}]^+$	D_1V_5	504	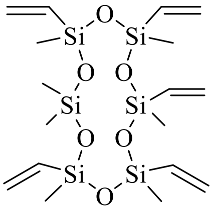	-
21	501 $[Si_6O_6C_{17}H_{33}]^+$	V_6	516	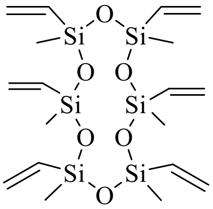	320
22	503 $[Si_7O_7C_{13}H_{39}]^+$	D_7	518	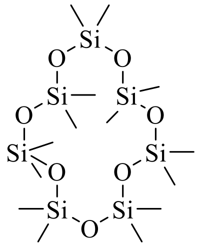	336
23	515 $[Si_7O_7C_{14}H_{39}]^+$	D_6V_1	530	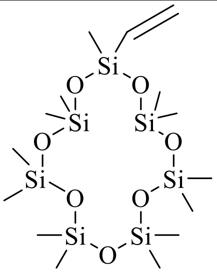	-
24	527 $[Si_7O_7C_{15}H_{39}]^+$	D_5V_2	542	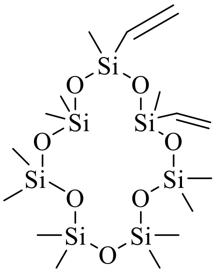	-

Table S2. *Detailed composition of cycles*

№	$MeVinSi(OMe)_2$/ $Me_2Si(OEt)_2$	Characteristics																									
		Composition of cyclosiloxanes, wt.%																									
		D_3	D_4	D_5	D_6	D_{n+1}	3 links		4 links			5 links				6 links					7 links		V_3	V_4	V_5	V_6	V_{n+1}
							D_2V_1	D_1V_2	D_3V_1	D_2V_2	D_1V_3	D_4V_1	D_3V_2	D_2V_3	D_1V_4	D_5V_1	D_4V_2	D_3V_3	D_2V_4	D_1V_5	D_6V_1	D_5V_2					
1	100/0	-	-	-	-	-	-	-	-	-	-	-	-	-	-	-	-	-	-	-	-	-	-	6,0	76,0	15,5	2,0
2	25/75	*	17,5	6,0	1,5	*	*	*	31,0	15,5	2,5	10,0	7,0	2,0	*	2,5	2,0	*	*	-	*	*	-	-	-	-	-
3	50/50	*	3,5	*	*	-	*	1,5	19,5	30,0	17,5	4,0	10,0	6,5	3,0	*	*	1,0	*	*	-	*	-	-	*	-	-
4	75/25	-	-	-	-	-	-	1,5	2,5	15,0	40,0	-	-	3,0	3,0	-	*	1,5	2,5	2,5	-	-	-	23,0	5,5	-	-
5	100/0	-	-	-	-	-	-	-	-	-	-	-	-	-	-	-	-	-	-	-	-	-	*	6,0	72,5	18,5	2,0
6	25/75	*	17,5	8,0	1,5	*	-	-	23,5	16,5	4,0	12,5	8,0	2,5	*	2,5	2,0	*	-	-	*	*	-	-	-	-	-
7	50/50	-	3,0	*	*	-	-	*	18,0	32,5	20,5	4,0	9,5	5,0	1,0	*	*	*	*	-	-	-	-	3,5	*	-	-
8	75/25	-	-	-	-	-	-	-	4,5	17,5	35,5	-	-	3,5	4,5	-	-	1,5	2,5	1,5	-	-	-	19,5	9,5	-	-

$* \leq 1$
